# First person – Jimena Monzón-Sandoval

**DOI:** 10.1242/dmm.049907

**Published:** 2022-10-18

## Abstract

First Person is a series of interviews with the first authors of a selection of papers published in Disease Models & Mechanisms, helping researchers promote themselves alongside their papers. Jimena Monzón-Sandoval is first author on ‘ Lipopolysaccharide distinctively alters human microglia transcriptomes to resemble microglia from Alzheimer's disease mouse models’, published in DMM. Jimena is a research associate in bioinformatics in the lab of Caleb Webber at Cardiff University, Cardiff, UK, investigating transcriptomics of induced pluripotent stem cell (iPSC)-derived brain cell types to model neurodegenerative diseases.


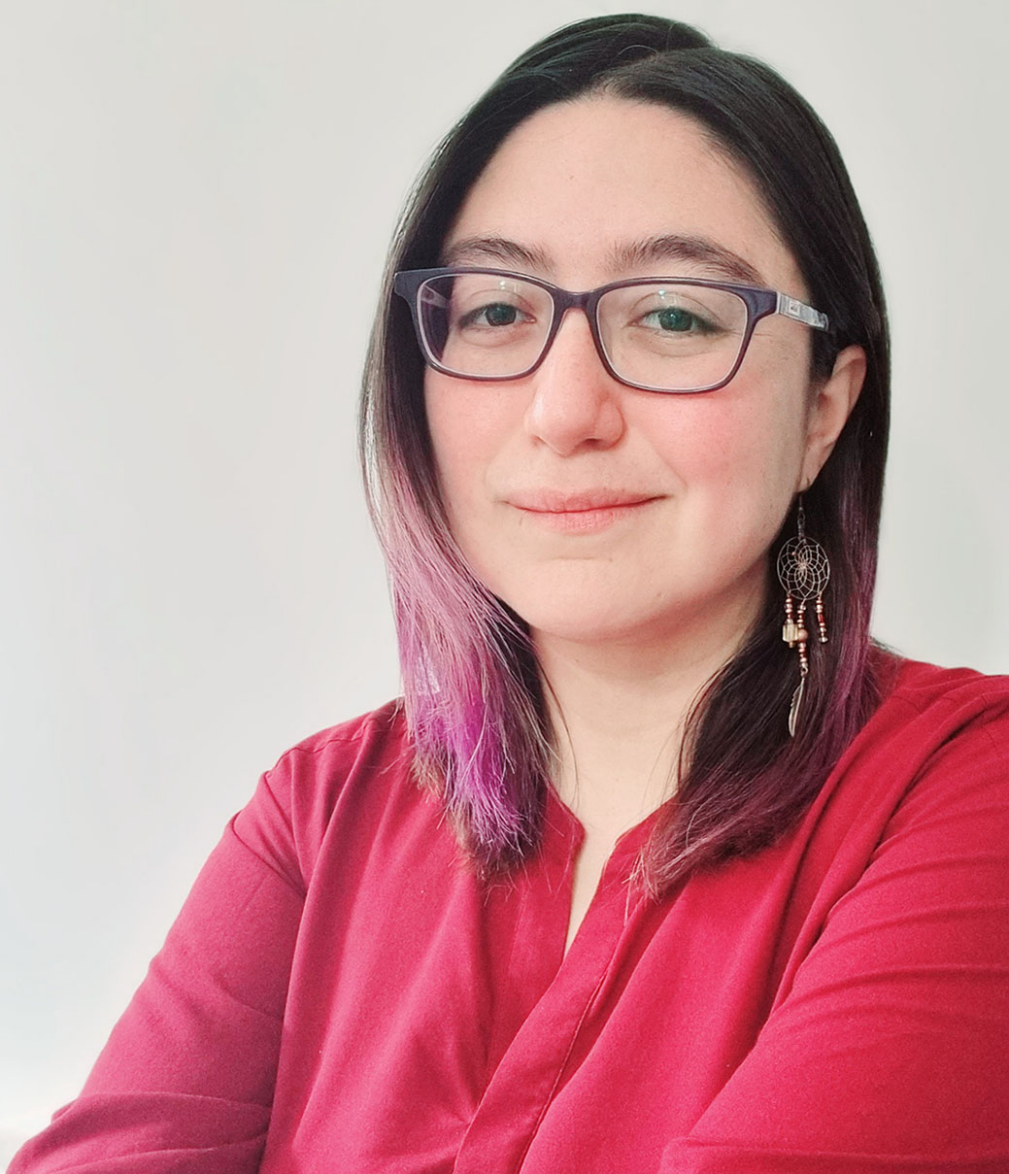


**Jimena Monzón-Sandoval**

**How would you explain the main findings of your paper to non-scientific family and friends?**

Linking the differences between us in our DNA to the chances of each of us getting Alzheimer's disease (AD) has picked out genes that act within a specific brain cell type called microglia as somehow involved in our risk of getting AD. These microglial cells are known as the immune cells of the brain. In this study, we derived cells that were similar to microglia and exposed those cellular models to different immune challenges to see if these immune challenges could cause these microglia models to react in a way that appeared to be similar to how microglia react in AD. Although the ways microglia models reacted to different stimulations were in some ways similar, we noticed that only their response to the stimulus lipopolysaccharide (LPS)+interferon gamma (IFN-γ) was similar to the gene expression changes that occur in microglia across several genetic mouse models of AD.

**What are the potential implications of these results for your field of research?**

The combination of LPS+IFN-γ is already a widely used stimulus in the field; what we uncover in our study is that only LPS+IFN-γ, and not ATPγS or prostaglandin E2 (PGE_2_), induces iPSC-derived microglia (iPSC-microglia) to a gene expression profile similar to that of various genetic mouse models of AD. By re-analysing publicly available data, we also observed the same effect in primary mouse microglia after LPS treatment. Taken together, our results indicate that LPS was the most relevant model for understanding AD (from the stimuli tested) as compared to several genetic mouse models of AD.

**What are the main advantages and drawbacks of the experimental system you have used as it relates to the disease you are investigating?**

As an iPSC model, iPSC-microglia allow us to study either naturally occurring genetic variation from the human population (from different donors) or particularly targeting a single gene while maintaining the same genetic background (isogenic). One of the main advantages of using iPSC-microglia is that we have an actual human model that is highly efficient and with relatively fast generation time. As some of the AD-associated genes (identified through genome-wide association studies) do not have a high similarity to their counterparts in mouse and potentially lead to a functional difference between species, using a human model is highly desirable. In combination with single-cell RNA sequencing, we were able to focus on particular cell populations while removing fibroblast-like and cycling microglial populations. One of the drawbacks is that it is an *in vitro* system, and wide differences exist between *in vitro* and *in vivo* microglia. Another concern of using iPSC-microglia relates to their maturity. Higher maturity could be reliant on external cues to which iPSC-microglia are not necessarily exposed *in vitro*. Despite these differences, we were able to identify a stimulus (LPS) that better reflects the changes observed in purified microglia from AD genetic mouse models.

“We identified a small […] set of genes that change their expression in response to the various stimuli in the iPSC-microglia and that have been previously identified to show gene expression changes in AD patients compared to healthy controls.”

**Figure:**
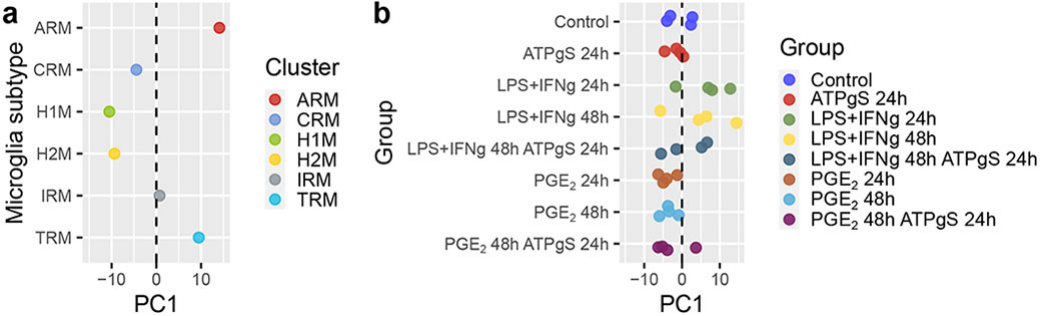
**The uncovered disease axis across diverse mouse microglia models (PC1) was able to segregate the transcriptional profiles of (a) previously known microglia subtypes identified in the *App^NL-G-F^* mouse and (b) iPSC-derived microglia treated with LPS+IFN-γ.** ARM, activated response microglia; CRM, cycling/proliferating microglia; H1M, homeostatic microglia 1; H2M, homeostatic microglia 2; IRM, interferon response microglia; TRM, transit response microglia.

**What has surprised you the most while conducting your research?**

We identified a small (but more than expected) set of genes that change their expression in response to the various stimuli in the iPSC-microglia and that have been previously identified to show gene expression changes in AD patients compared to healthy controls. However, most gene expression changes in this comparison were surprisingly in the opposite direction; this discrepancy remains to be further investigated. Another remarkable surprise was how well the transcriptional profiles of microglia subtypes fitted within the disease axis we uncovered using a data-driven approach. We encountered a neat way to segregate homeostatic from activated response microglia, which was quite reassuring about the validity of our approach.

**What do you think is the most significant challenge impacting your research at this time and how will this be addressed over the next 10 years?**

One significant challenge relates to the time and space in which we study disease. In terms of time, in human we either have relatively immature iPSC-derived models or nuclei from human post-mortem tissue, with still very limited access to fresh tissue. While there is considerable progress in creating more mature iPSC-derived cell types that better resemble their body counterparts, further improvement of differentiation and maturation strategies could be key to accelerating the discovery of disease mechanisms. Some directions that have been explored are transplanting iPSC-derived cells to *in vivo* models (like mouse), or the use of co-cultures and organoids composed of diverse iPSC-derived cell types, with the drawback of increased heterogeneity. In terms of space, relatively new technologies like spatial transcriptomics are already useful to focus on regions of interest, either for the study of the cell in a particular microenvironment or the interactions between different cell types. In general, in several neurodegenerative diseases, we still have a great challenge to pick apart small or very small effects of variants associated with a particular disease. Reducing and/or accounting for unwanted variability in our models and experimental designs could help us to distinguish those small effects. Alternatively, the use of simultaneous gene targeting could help us understand the function/dysfunction of interacting genes with minor effects.

“Early opportunities for undergrads to be involved in research projects can make a difference.”

**What changes do you think could improve the professional lives of scientists?**

Early opportunities for undergrads to be involved in research projects can make a difference. During my undergrad, I had the opportunity to do a few internships in very diverse research labs. It was important not only to gain knowledge/techniques from different fields but also to learn to work with different people. During my PhD, one of my supervisors particularly encouraged me to apply for travel grants and present my work to diverse audiences. It is never too early to start to get your own funding – even if it’s a small amount, it builds up. Full scholarships and better funding schemes are still needed so everyone would be able to dedicate full time to do the science during a PhD. Now, as a postdoctoral researcher and particularly as a bioinformatician, all the work I do is through collaborations. I find dedicated networking opportunities with peers particularly valuable, although having a mentor guidance though the different steps of the academic career is also important. In terms of mental health, there has been an increased awareness of its importance, but we still need to improve job security. I am lucky to have a very supportive PI that has been able to secure funding for me though the last few years, but unfortunately the norm is to work on short fixed-term contracts. Finally, at any stage, but particularly at PI level, I am sure that less administrative work would be beneficial.

**What's next for you?**

First, I would like to keep learning new things and dedicating my time to research. For example, now I am excited to start working with spatial transcriptomic data. I have been a postdoctoral researcher for a while now, so next would be to get my own funding.
